# Challenges in Investigating Psychotic-Like Experiences in an Adolescent Awaiting an Autism Assessment

**DOI:** 10.1192/bjo.2024.681

**Published:** 2024-08-01

**Authors:** Jade Parkinson

**Affiliations:** West London NHS Trust, London, United Kingdom

## Abstract

**Aims:**

Studies estimate that 90% of people with a diagnosis of autism experience sensory abnormalities. The majority of those affected will not have a psychotic illness, however young people with autism are three to six times more likely to develop schizophrenia than their neurotypical equivalents.

This report considers the diagnostic complexities, potential risks and challenges of navigating concurrent referral and treatment pathways for an adolescent awaiting an autism assessment, who has psychotic-like experiences.

**Methods:**

An adolescent female was referred to our Tier 3 service for an autism assessment. Whilst on the waiting list, our service was contacted on three occasions by adults who knew the patient, expressing concerns that she had psychotic-like experiences, namely perceptual abnormalities which had not been included in the original referral.

On the third occasion, approximately six months after the initial referral was accepted, a decision was made to review the patient face-to-face to explore these symptoms further.

During this review she appeared to have positive and negative symptoms of schizophrenia, including perceptual abnormalities in all sensory modalities, thought block, paranoid ideation and a mood incongruent affect. Her sleep cycle was reversed and she had not attended school for several years.

She was subsequently referred to the Early Intervention Psychosis Service, underwent an eight week assessment and was discharged back to the autism service.

**Results:**

Young people in the UK are on average waiting nine months for an autism assessment, although some are waiting up to seven years for treatment. NICE recommends that young people referred due to first episodes of psychosis are seen within two weeks, as delays in treatment can negatively impact on the patient's response to treatment.

Diagnostic uncertainty can arise due to overlapping symptoms, clinician inexperience and difficulties with eliciting a thorough history. With waiting times for autism assessments growing, young people who may have psychotic symptoms are waiting longer to see a clinician. The referral pathways for neurodevelopmental and psychiatric disorders typically exist independently, but inclusion on one pathway can create barriers in accessing the other.

**Conclusion:**

It is good practice for comorbid psychiatric disorders to be considered by the referrer, when referring a young person for an autism assessment.

Clinicians should avoid making assumptions regarding the aetiology of symptoms based on the original reason for referral, explore symptoms thoroughly and refer to alternative services if needed.

